# Disrupted Subcortical-Cortical Connections in a Phonological but Not Semantic Task in Chinese Children With Dyslexia

**DOI:** 10.3389/fnhum.2020.611008

**Published:** 2021-01-18

**Authors:** Lihuan Zhang, Jiali Hu, Xin Liu, Emily S. Nichols, Chunming Lu, Li Liu

**Affiliations:** ^1^State Key Laboratory of Cognitive Neuroscience and Learning & IDG/McGovern Institute for Brain Research, Beijing Normal University, Beijing, China; ^2^Faculty of Education, Western University, London, ON, Canada; ^3^Brain and Mind Institute, Western University, London, ON, Canada

**Keywords:** Chinese reading disability, subcortical regions, fMRI, functional connectivity, graph theory

## Abstract

Reading disability has been considered as a disconnection syndrome. Recently, an increasing number of studies have emphasized the role of subcortical regions in reading. However, the majority of research on reading disability has focused on the connections amongst brain regions within the classic cortical reading network. Here, we used graph theoretical analysis to investigate whether subcortical regions serve as hubs (regions highly connected with other brain regions) during reading both in Chinese children with reading disability (*N* = 15, age ranging from 11.03 to 13.08 years) and in age-matched typically developing children (*N* = 16, age ranging from 11.17 to 12.75 years) using a visual rhyming judgment task and a visual meaning judgment task. We found that the bilateral thalami were the unique hubs for typically developing children across both tasks. Additionally, subcortical regions (right putamen, left pallidum) were also unique hubs for typically developing children but only in the rhyming task. Among these subcortical hub regions, the left pallidum showed reduced connectivity with inferior frontal regions in the rhyming judgment but not semantic task in reading disabled compared with typically developing children. These results suggest that subcortical-cortical disconnection, which may be particularly relevant to the phonological and phonology-related learning process, may be associated with Chinese reading disability.

## Introduction

Recently, an increasing number of studies have reported that subcortical regions are involved in the language and reading neural network (Tomasi and Volkow, 2012; Hebb and Ojemann, 2013; Liu et al., 2018; Tang et al., 2018; also see in a review: Price, 2012). For example, a resting-state functional connectivity study including 970 healthy subjects found that Broca's and Wernicke's areas connect not only classic cortical regions engaged in language processing (i.e., pre-frontal, temporal, and parietal regions) but also to subcortical regions (i.e., bilateral caudate, left pallidum, left putamen, and left thalamus) (Tomasi and Volkow, 2012). Further, previous studies have shown that thalamus and basal ganglia are involved in multiple language-related processes such as phonological processing (Booth et al., 2007b; Hebb and Ojemann, 2013), speech production (Bohland and Guenther, 2006; Tang et al., 2018), orthographic processing (Braun et al., 2019), lexical semantic processing (Kotz et al., 2002; Ketteler et al., 2008), as well as non-linguistic processes, such as procedural learning (Schultz et al., 1997; Ullman, 2004; Shohamy et al., 2008; Schreiweis et al., 2014). For example, the left putamen was reported to be activated in a visual rhyming judgment task and was thought to be involved in the initiation of the phonological processing (Booth et al., 2007b). Bohland and Guenther (2006) found that bilateral putamen involved in overt speech production and bilateral anterior thalamus and caudate were related to sequence complexity during overt speech production. And the left pallidus and the left caudate were found to be more activated when recognizing words with higher frequency and more similar-orthographic neighbors in a silent reading task, suggesting these regions engage in the matching of orthographic language input onto the representations in long-term memory (Braun et al., 2019). Pugh et al. (2013) found that activation of the left thalamus was significantly correlated with reading scores in children, and they suggested that future studies investigating reading and reading disorders should include both cortical and subcortical regions. Taken together, these studies suggest that subcortical structures play an important role in language processing.

Developmental dyslexia is a neurological disorder, in which individuals have difficulty acquiring fluency in reading, despite having normal intelligence and motivation (Lyon et al., 2003; Shaywitz and Shaywitz, 2005). Many studies have also found that atypical brain function in individuals with dyslexia in alphabetic scripts includes not only cortical brain regions (see in meta-analysis: Maisog et al., 2008; Richlan et al., 2009; Paulesu et al., 2014; Martin et al., 2016) but also some subcortical regions, such as hypoactivation in the left thalamus (Gaab et al., 2007; Díaz et al., 2012) and hyperactiveation in the bilateral caudate (Hoeft et al., 2007; Richlan et al., 2011 only in the left caudate) and right thalamus (Hoeft et al., 2007). A recent review of neurobiological studies in children with language impairment and dyslexia suggested that dysfunction of corticostriatal systems, which may impact language learning processes due to impairment in extracting and decoding complex rules, may be an alternative cause for dyslexia aside from the dysfunction of the cortical system for phonological representation and manipulation (Krishnan et al., 2016).

The prevalence of dyslexia ranges from 5 to 8% in Chinese school-aged children (Stevenson et al., 1982; Zhang et al., 1996). Similar to research on alphabetic dyslexia, research on Chinese dyslexia has also reported dysfunction in subcortical regions, including the left putamen (Siok et al., 2008; Wang et al., 2019) and left thalamus (Siok et al., 2008), although most studies (Siok et al., 2004, 2008; Liu et al., 2012, 2013) have mainly reported brain abnormality in cortical brain regions. Specifically, Siok et al. (2008) found reduced activation in left putamen and thalamus in Chinese dyslexic children compared to age-matched controls when performing a visual character rhyming judgment task. Wang et al. (2019) found reduced gray matter volume and altered functional abnormality of the putamen in Chinese dyslexic children. All of these above studies have indicated that abnormal subcortical regions may be associated with or be a cause of reading impairment.

Previous brain connectivity studies have further suggested that alphabetic dyslexia is a disconnection syndrome (Horwitz et al., 1998; Stanberry et al., 2006; van der Mark et al., 2011; Vogel et al., 2012; Boets et al., 2013; Koyama et al., 2013; Schurz et al., 2015; Morken et al., 2017), and this is also true for Chinese dyslexia (Zhou et al., 2015; Cao et al., 2018; Wang et al., 2019). Particularly relevant to the current study, Zhou et al. (2015) found altered resting functional connectivity between left middle frontal and visual-orthographic processing regions (i.e., left visual word form area and left intraparietal sulcus) in Chinese children with dyslexia compared to age-matched controls (Zhou et al., 2015). In another study, lower connectivity was found between the left superior temporal gyrus and left fusiform gyrus in Chinese children with dyslexia than both age-matched controls and reading-matched controls in an auditory rhyming task, suggesting a weak connection between phonology and orthography (Cao et al., 2017). In addition, Cao et al. (2018) found that Chinese children with dyslexia showed weaker functional connectivity between the left inferior frontal gyrus (a phonological processing region) and the left middle occipital gyrus (an orthographical processing region) compared to age- and reading-matched controls in a visual orthography judgment task. However, these functional connectivity studies of Chinese dyslexia focused on the connectivity within the classic cortical reading network. To our knowledge, only one study (Wang et al., 2019) has reported altered functional connectivity between the left putamen and cortical regions relative to age- and reading-matched children in an auditory rhyming task, suggesting that an abnormal subcortical-cortical connectivity pattern may be a contributing factor to Chinese dyslexia.

Although a small number of studies (Siok et al., 2008; Wang et al., 2019) have reported abnormal brain structure, brain activation and functional connectivity in subcortical regions in Chinese dyslexia during language or reading tasks in Chinese children with dyslexia, the role of subcortical regions in reading and reading impairment is less understood compared with that of the cortical brain regions. There is a need to systematically evaluate the role of subcortical regions in typical as well as atypical reading development. Toward this aim, the current study used a graph theoretical approach to examine whether and which subcortical regions are hubs (that is, a key region connecting many other brain regions) in the brain network for reading in typically developing (TD) Chinese children and reading disabled (RD) Chinese children. Additionally, we sought to address whether hub distribution differed between groups, particularly within subcortical regions. Our third aim was to examine how these subcortical hubs interact with the classic cortical brain regions involved in reading, and whether group differences exist in the subcortical-cortical connections. Specifically, we adopted a visual rhyming judgment and a visual meaning judgment task to examine the connectivity of subcortical regions in the brain networks of phonological and semantic processing during Chinese character reading. We predicted that subcortical regions serve as hub in the reading process similar to cortical regions of typical readers, particularly in the phonological processing task as previous studies have implicated subcortical regions in phonology-related (Booth et al., 2007b; Hebb and Ojemann, 2013) and speech articulation (Bohland and Guenther, 2006; Kita et al., 2013; Tang et al., 2018) processes. Additionally, subcortical regions, particularly the striatum, play a role in procedural learning (Schultz et al., 1997; Ullman, 2004; Shohamy et al., 2008; Schreiweis et al., 2014), and learning phonology may involve more procedural processes than learning the meaning of a character. Finally, because previous work has shown altered putamen-cortical connectivity in Chinese children with dyslexia (Wang et al., 2019), we predicted that functional connectivity between subcortical regions and the cortical reading network would be altered in the RD group during the visual rhyming judgment task.

## Materials and Methods

### Participants

Sixteen typically developing (TD) children (5 females; age: mean = 11.79 years, SD = 0.39) and 15 children with reading disability (RD) (5 females; age: mean = 12.06 years, SD = 0.49) participated in this study. Amongst the participants, some (11 TD children and 11 RD children) came from a previously published study (Liu et al., 2012) by the same research group, and the other subjects (5 TD children, 4 RD children) were new participants. This study was approved by the Institutional Review Board at Beijing Normal University. Informed consent was obtained from all children and their parents. The children were included according to the following criteria: (1) native Chinese speakers; (2) right-handed; (3) normal or corrected-to-normal vision and normal hearing; (4) no psychiatric or neurological disease; (5) not taking medication that influenced the central nervous system; and (6) not having attention deficit hyperactivity disorder.

Non-verbal intelligence and reading ability were measured in all children who met these criteria. Children's non-verbal intelligence was measured using the Chinese version of the Wechsler Intelligence Scale for Children-Revised (WISC-R) (Wechsler, 1974) and scored by local norms (Lin and Zhang, 1986). Reading ability was measured by the Character Recognition Measure and Assessment Scale for Primary School Children (CRM) (Wang and Tao, 1993) and the character reading fluency (CRF) test (Liu et al., 2013). The CRM test measures how many characters a child can recognize among 3,500 of the most commonly used Chinese characters. CRM is a widely used test to screen dyslexic Chinese children (Liu et al., 2012, 2013; Qi et al., 2016). Here, we used the CRM test score as a criterion for screening children with reading disability. The CRF is a single-character reading fluency test. It comprises 135 high-frequency Chinese characters chosen from primary school textbooks. The characters are sorted into columns and ordered from easy to difficult. Children are asked to read aloud the characters sequentially, column by column, as fast and correctly as possible. The number of characters read correctly and the time participants spent are recorded. The speed (correct number/time) is used as the test score. Here, CRF was used as an index for reading fluency.

All children had a normal non-verbal IQ (above 90). Children whose character recognition scores tested by CRM were 1.5 years below the average level of the same age were included in the RD group. Children whose scores were not 1.5 years below their age level were included in the TD group. Demographic information and task performance of all children (16 in the TD group and 15 in the RD group) are shown in Table 1. The two groups were matched in age (*t*(29) = −1.717, *p* = 0.097), sex (Mann-Whitney *U* = 117.5, *p* = 0.900), and PIQ (*t*(29) = 1.748, *p* = 0.091). The RD group scored significantly lower on the CRM (*t*(29) = 9.759, *p* < 0.001) and CRF (*t*(29) = 6.803, *p* < 0.001) than the TD group. Data from one child in the RD group in the rhyming judgment task were excluded due to large head motion artifacts (larger than 3 mm). As a result, the following data analysis included 14 RD children in the rhyming judgment task. The group differences in demographics and reading performance remained similar after the one RD child was excluded (more details are shown in Supplementary Table 1).

**Table 1:** Demographics and task performance of all the typically developing (TD) and reading disabled (RD) children.

	**TD**	**RD**	***p***
Sex (M/F)	11/5	10/5	0.900
Age [mean (SD)]	11.78(0.39)	12.06(0.49)	0.097
Performance IQ	111(15.0)	103(9.7)	0.091
CRM	3031(150)	2550(98)	<0.001
CRF	1.12(0.18)	0.60(0.14)	<0.001
Rh_ACC [mean (SD)]	73% (0.12)	58% (0.09)	<0.001
Rh_RT [mean (SD)]	1755(445)	1361(381)	0.013
Me_ACC [mean (SD)]	82% (0.10)	73% (0.11)	0.013
Me_RT [mean (SD)]	1492(394)	1417(323)	0.570

*TD: typically developing group, RD: reading disabled group, CRM: Character Recognition Measure and Assessment Scale for Primary School Children, CRF: character reading fluency test. Rh_ACC and Rh_RT: the accuracy and reaction time (ms), respectively, in the character condition of the rhyming judgment task; Me_ACC and Me_RT: the accuracy and reaction time, respectively, in the character condition of the meaning judgment task*.

### Design and fMRI Paradigm

This study is a re-analysis of part of the data from a previously published study (Liu et al., 2012). The previously published study used traditional brain activation analysis to reveal the neural abnormalities of Chinese children with reading disability. However, the current study used a graph theoretical approach to examine the functional connectivity of subcortical regions. A rhyming judgment task and a meaning judgment task were used to investigate the two key processes of reading: orthography-to-phonology (O-P) mapping and orthography-to-semantics (O-S) mapping. Two Chinese characters were displayed on the center of the screen sequentially. Each character was displayed for 800 ms with a 200 ms interval between the two characters. Then, a red fixation cross was presented to remind the participants to respond. The duration of the red cross was jittered among 2,200, 2,600, and 3,000 ms with equal probability. Participants were asked to judge whether the two characters were rhyming or not in the rhyming judgment task and whether they were semantically related or not in the meaning judgment task. When two characters rhymed in the rhyming judgment task or were semantically related in the meaning judgment task, participants were asked to press a button with their right index finger; otherwise, they were asked to press another button with their right middle finger. In both tasks, half of the pairs presented in trials were rhyming or related, and half were not. Characters in both tasks were chosen from textbooks for primary school and were matched for frequency, acquisition age, and number of strokes. More detailed information about the materials could been found in the previous paper (Liu et al., 2012).

In addition to the character condition above, there were two control conditions for each task. These two conditions were designed to exclude brain activation due to basic visual processing, decision-making processing and motion execution processing. The two conditions were perceptual control and null control. In the perceptual control condition, participants were asked to determine whether two sequentially presented Tibetan symbols were identical or not. Half of the pairs presented in the perceptual condition were identical. In the null control condition, two black crosses were sequentially presented, and participants were asked to press a button with their right index finger when the second cross turned blue. The procedures and timing for these two conditions were the same as those for the character condition.

We used an event-related design in our experiment. There were four runs in total (two runs for each task). Each run was 6 min 44 s in length. Forty-eight pairs of character stimuli, 12 pairs of perceptual stimuli and 24 pairs of null stimuli were presented in each run. Instructions were promptly provided before each run to brief the participants about the task. In addition, there was a 12 s period at the beginning to acquire an equilibrium magnetization of the scanner and a 22 s period at the end to allow deconvolution of the entire hemodynamic response function (HRF) of the last trial.

### Image Acquisition

Imaging data were acquired using a 3T Siemens scanner (MAGNETOM Trio, a Tim System) at Beijing Normal University. The susceptibility weighted single-shot echo planar imaging (EPI) method was used to obtain blood oxygenation level-dependent (BOLD) images during the task. The following scan parameters were used: TR = 2,000 ms; TE = 20 ms; flip angle = 80°; number of slices = 32; slice thickness = 3 mm, gap = 0.48 mm; FOV = 220 ^*^ 206 mm; matrix = 128 ^*^ 120 ^*^ 32; and voxel size = 1.72 ^*^ 1.72 ^*^ 3.48 mm. In addition, we also acquired a structural T1 weighted 3D image (MPRAGE) with the following parameters: TR = 2,300 ms; TE = 3.36 ms; number of slices = 160; slice thickness = 1 mm; FOV = 256 ^*^ 256 mm; matrix = 256 ^*^ 256 ^*^ 160; and voxel size = 1 ^*^ 1 ^*^ 1 mm.

Before formal imaging acquisition, the children were first put in a simulated scanner to get used to the scanning environment. They then completed a short practice session for each of the two tasks to help them become familiar with the experiment instructions and procedures.

### Data Pre-processing

The imaging data was preprocessed using Data Processing Assistant for Resting-State fMRI (DPARSF)^1^. First, we deleted the first 10 volumes of each participant's functional imaging data to ensure the steadiness of the signal. We then performed slice-timing to correct the scanning time delay. Realignment was then used to correct head motion during scanning. After excluding the participants whose head motion was larger than 3 mm, we coregistered each participant's T1 structural image to their mean functional images. Next, we segmented and normalized the coregistered T1 images to Montreal Neurological Institute (MNI) space. The functional images were normalized to a 3 ^*^ 3 ^*^ 3 mm^3^ voxel size. The resampled functional images were smoothed with an 8 mm FWHM (full width at half maximum) Gaussian filter. Detrending with a high-pass filter (0.01–0.08 Hz) was also applied to the images. Finally, we regressed out the six head-motion parameters, cerebrospinal fluid signal, and white mater signal.

### Graph Theory and Network Construction

Graph theory is an effective method to investigate interregional connectivity networks within the whole brain. Graph theory treats the structural or functional brain network as a graph that is composed of nodes (brain regions) and edges (structural or functional connections) (Bullmore and Sporns, 2009, 2012). Previous studies using this method have reported many novel findings in terms of the alterations in the structural (Hosseini et al., 2013; Liu et al., 2015; Qi et al., 2016) and functional (Finn et al., 2014; Edwards et al., 2018; Yang and Tan, 2020) brain networks of children with dyslexia.

Network construction and network property calculations were processed using Graph Theoretical Network Analysis Toolbox (GRETNA)^2^. We used the same network construction method as a previously published paper (Liu et al., 2018). Briefly, according to the Automated Anatomical Labeling (AAL) tool (Tzourio-Mazoyer et al., 2002), we first divided the whole brain (excluding the cerebellum) of each participant into 90 regions. For each region, the mean time series of all voxels within this region was used as the regional time series. Then, we extracted 2 TR time series after the onset of the first stimulus in each trial individually for 90 regions of each participant for both tasks separately. We chose the current duration because the duration from the onset of the first stimulus to the end of the trial was about 4.4 s in average in each trial. Next, we concatenated the trials for the three conditions separately in each task (character condition, perceptual condition and null condition) (Ekman et al., 2012). As a result, we obtained time series data for 90 regions for each participant in each of the three conditions of the two tasks separately.

To construct an interregional correlation network, we then calculated Pearson's correlation coefficient between each pair of regional time series for each participant, for each condition, for each task. As a result, each participant had six 90^*^90 connectivity matrices (interregional correlation network). Binary matrices were then generated from the 90^*^90 connectivity matrices of each participant using different sparsity threshold values. If the absolute value of the correlation coefficient was above the threshold, then its value in the binary matrix was set to 1; otherwise, its value was set to 0. We applied a series of sparsity thresholds (from 0.05 to 0.4, with a step size of 0.01) to ensure that the networks were fully connected and still maintained small-world architecture (Achard et al., 2006; Liang et al., 2016). The networks were not fully connected at lower sparsity values (<0.05) and were less likely to maintain small-world architecture at higher sparsity values (>0.4).

### Network Properties Calculation

The network properties of the functional brain binary networks we obtained above were characterized using graph theoretical measures. As the major aim of the current study was to explore the role of the subcortical regions in typical as well as atypical reading development, we concentrated on the nodal properties of the subcortical nodes. The nodal degree of a particular node is the number of direct connections with other nodes in the whole network. A high nodal degree indicates that the node is highly connected in the network. The nodes whose normalized degrees are 1 SD above the mean degree of all nodes in the network are defined as hubs.

### Statistical Analysis

#### Hub Distribution Analysis

We identified the hubs for each group in each task and classified them as group-shared or group-unique hubs. Because we applied a series sparsity threshold to obtain the nodal degree, the degree changed when the threshold changed. Therefore, we calculated the area under the curve (AUC) to obtain the index of nodal topology, which was independent of the threshold. Then, we transformed the degree into a *Z* score using the mean and standard deviation of all nodes. Nodes whose degrees were 1 SD above the mean were identified as hubs. Using the method above, we obtained a list of hubs for each group per task. We excluded the hubs that were also identified as hubs in the two control conditions.

#### Inter-Regional Correlation Analysis

We first examined how the subcortical hubs interacted with the classic cortical reading regions in both tasks separately for each group by performing inter-regional correlation analysis. We then used two sample *t*-tests to explore whether these significant subcortical-cortical correlations for the TD or the RD group would significantly differ. Specifically, based on previous meta-analyses of Chinese reading (Bolger et al., 2005; Tan et al., 2005; Wu et al., 2012), we chose 16 cortical regions as the classic reading network, including regions in the left frontal lobe (i.e., middle frontal gyrus, the opercular part of inferior frontal gyrus and the triangular part of inferior frontal gyrus), the left parietal lobe (i.e., inferior parietal lobe and superior parietal gyrus), the left temporal lobe (i.e., inferior temporal gyrus, middle temporal gyrus, and superior temporal gyrus), and in the bilateral occipital lobe (i.e., inferior occipital gyrus, middle occipital gyrus, and lingual gyrus) and bilateral fusiform gyri. The interregional correlation coefficients were first transformed into normalized *z*-scores using the Fisher *z*-transformation before evaluating them via *t*-tests. We added age, the PIQ and the ACC of the task as covariates in the *t*-tests. A significance level of *p* < 0.05 corrected using a false discovery rate (FDR) was adopted in group comparisons. In order to validate our results, we further conducted a permutation test on the interregional correlations showing significant group difference in the *t*-test.

#### Brain-Behavior Correlation Analysis

In order to reveal the relationship between the altered subcortical-cortical connections and the deficits in reading process, we further calculated the Pearson's correlation coefficient between the task performance and the subcortical-cortical connections showing significant group difference for each group separately in each task. Before calculating the correlation coefficient, we firstly transformed the inter-regional correlation coefficients and the score of task performance into normalized z-scores using the Fisher *z*-transformation.

## Results

### Task Performance of the Two Groups

The RD group responded significantly faster(*t*(28) = −2.464, *p* = 0.020) but less accurate (*t*(28) = 3.819, *p* < 0.001) in the rhyming judgment task than in the TD group (Supplementary Table 1). As for the meaning judgment task, the accuracy of the RD group was significantly lower (*t*(29) = 2.660, *p* = 0.013) than the TD group, but there was no group difference the in the response time (Table 1).

### Hub Distribution of the Two Groups

**Figure 1:**
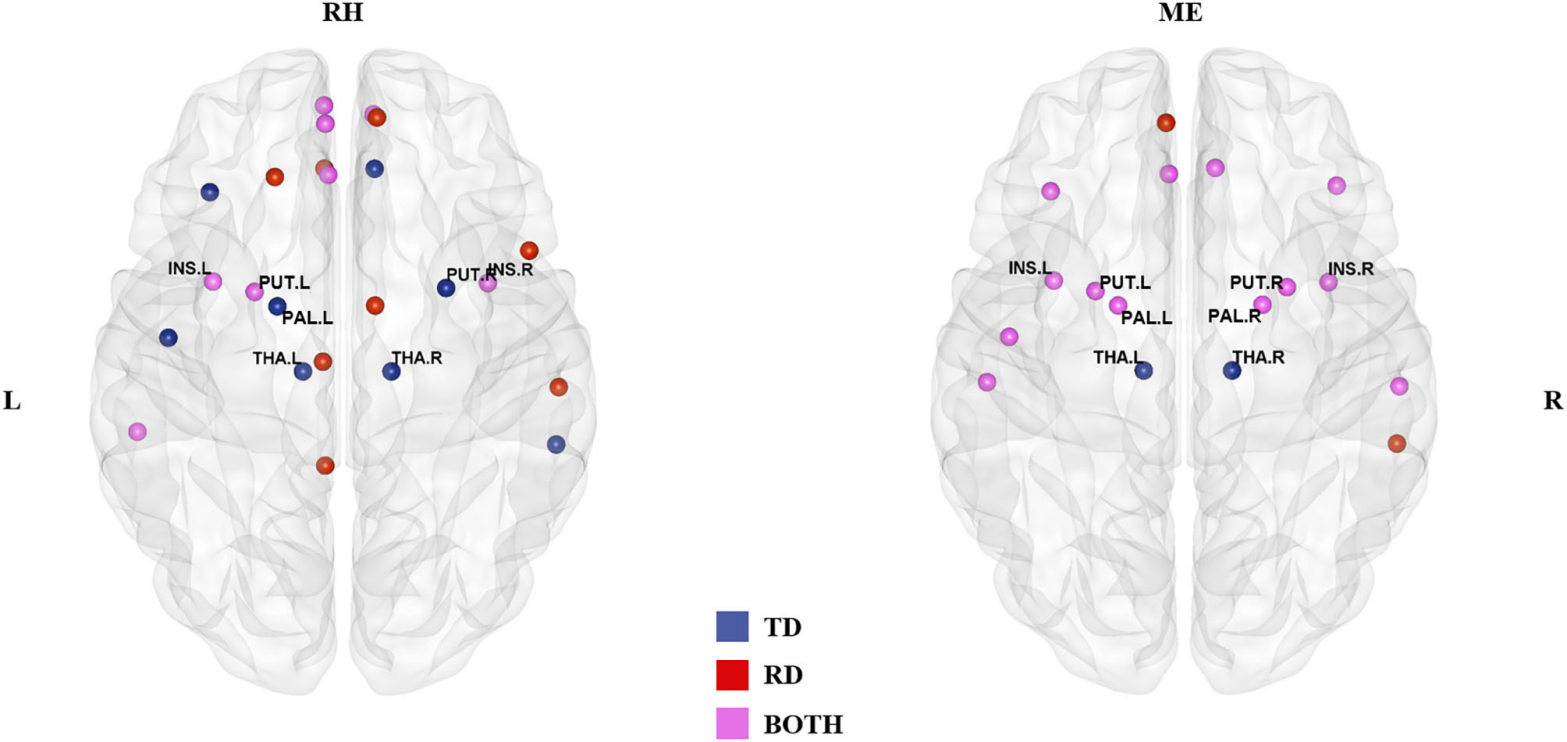
Hub distribution of the two groups in the rhyming judgment task (left) and in the meaning judgment task (right). Both groups had some subcortical regions as hubs. In addition, the two groups had more shared hubs in the meaning than the rhyming judgment task. TD, typically developing group; RD, reading disabled group; RH, rhyming judgment task; ME, meaning judgment task; L, left; R, right. See Abbreviations for full names of the abbreviations.

In the rhyming judgment task, 16 hubs were identified for each group, of which eight (33% overlapping rate) were shared (Table 2; Figure 1). The unique hubs in the TD group were mainly subcortical regions, including the bilateral thalami (THA) and the right putamen (PUT). In the RD group, the unique hubs were mainly regions in the frontal lobe, including the right medial superior frontal gyrus (SFGmed), left dorsolateral superior frontal gyrus (SFGdor), right opercular inferior frontal gyrus (IFGoperc), and left gyrus rectus (REC).

**Table 2:** Hub distribution for the two groups in the rhyming and meaning judgment task.

	**TD**	***Z* Score**	**RD**	***Z* Score**
*RH*	INS.L	2.482	SFGmed.L	1.742
	ACG.L	2.067	ORBsupmed.L	1.730
	PUT.L	2.026	ORBsupmed.R	1.665
	INS.R	1.874	INS.L	1.426
	ORBsupmed.R	1.343	MTG.L	1.354
	ORBsupmed.L	1.314	INS.R	1.348
	MTG.L	1.234	PUT.L	1.309
	SFGmed.L	1.068	ACG.L	1.034
	ACG.R	1.628	SFGmed.R	1.682
	PUT.R	1.548	SFGdor.L	1.416
	MTG.R	1.466	SMA.R	1.280
	THA.R	1.319	PCG.L	1.218
	PAL.L	1.316	IFGoperc.R	1.157
	ROL.L	1.209	STG.R	1.144
	ORBinf.L	1.031	DCG.L	1.032
	THA.L	1.011	REC.L	1.031
*ME*	INS.L	2.550	INS.L	2.175
	PUT.L	2.343	PUT.L	2.056
	PUT.R	1.813	ACG.R	1.982
	INS.R	1.764	ACG.L	1.949
	ACG.R	1.756	INS.R	1.938
	PAL.L	1.741	PUT.R	1.720
	ACG.L	1.656	ORBinf.L	1.416
	ROL.L	1.536	ORBinf.R	1.346
	ORBinf.L	1.361	STG.L	1.263
	STG.R	1.108	STG.R	1.173
	STG.L	1.100	PAL.L	1.168
	ORBinf.R	1.096	PAL.R	1.023
	PAL.R	1.061	ROL.L	1.009
	THA.R	1.228	MTG.R	1.263
	THA.L	1.094	SFGmed.L	1.104

*TD, typically developing group; RD, reading disabled group; RH, rhyming judgment task; ME, meaning judgment task; L, left; R, right. Regions above the “—” were hubs shared between the two groups. See Abbreviations for full names of the abbreviations*.

In the meaning judgment task (Table 2; Figure 1), there were 15 hubs for each group, 13 of which (76% overlapping rate) were shared. The unique regions in the TD group were again subcortical regions (bilateral THA), while the unique regions in the RD group were cortical regions (right middle temporal gyrus (MTG) and left SFGmed). The two groups shared more hubs in the meaning judgment task than in the rhyming judgment task (76 vs. 33%, respectively).

Focusing on subcortical regions (Supplementary Table 2), within the TD group the bilateral THA were identified as unique hubs in both tasks, and the right PUT and left pallidum (PAL) in the rhyming judgment task. There were no TD-unique subcortical hubs specific to the meaning judgment task, and no RD-unique subcortical hubs in either task. When examining subcortical hubs that were common between groups, several regions were identified. Specifically, both groups shared the bilateral insula (INS) and left PUT as hubs across tasks, while the bilateral PAL and right PUT were shared within the meaning judgment task. There were no shared subcortical hubs specific to the rhyming judgment task.

### Group Differences in the Subcortical-Cortical Inter-Regional Correlations

**Figure 2:**
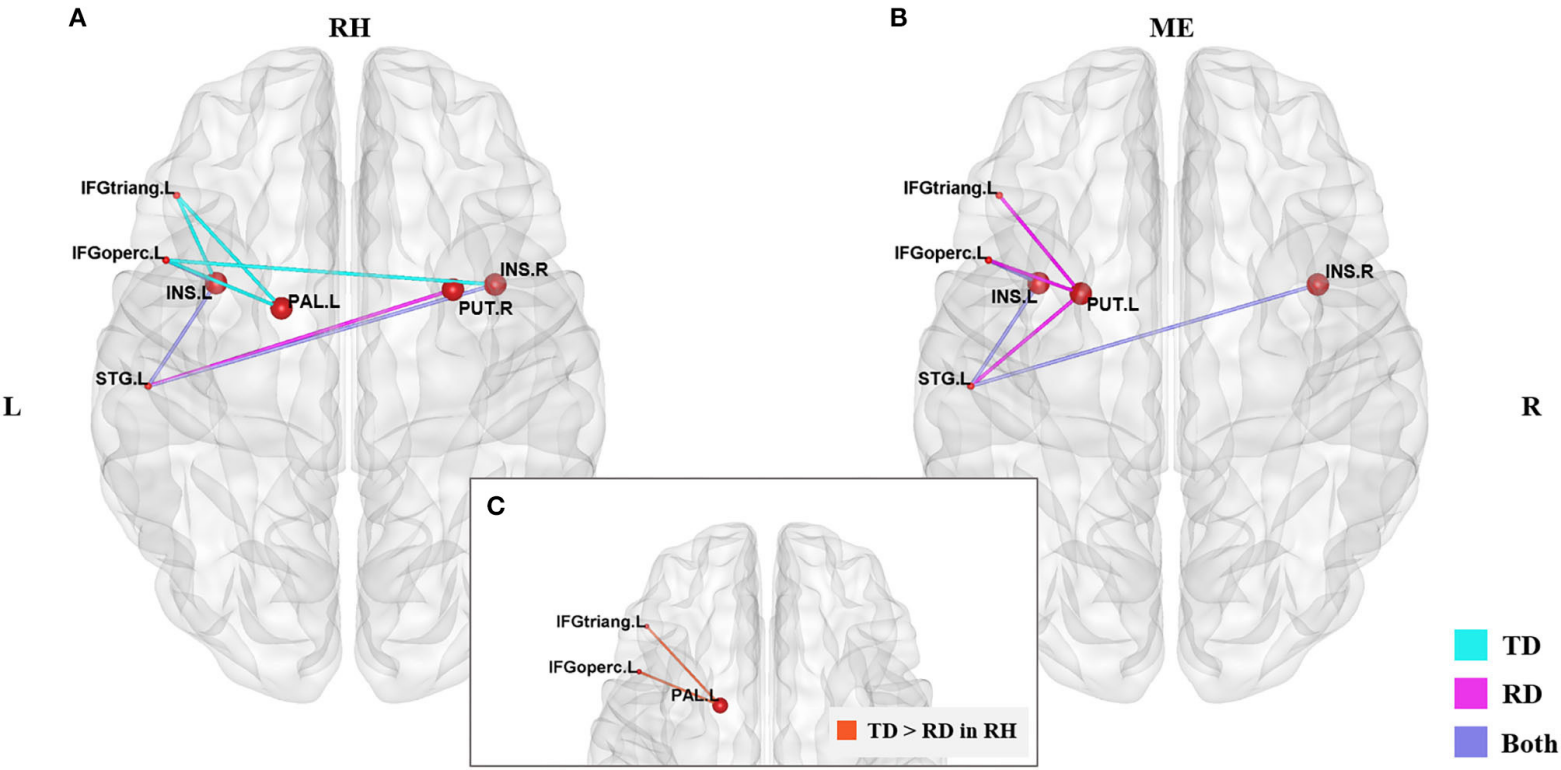
Significantly larger subcortical-cortical correlations in the TD than RD group were found in the rhyming judgment task. **(A)** Significant subcortical-cortical correlations of the two groups in the rhyming judgment task. **(B)** Significant subcortical-cortical correlations of the two groups in the meaning judgment task. Blue lines represent significant correlations in the TD group, pink lines represent the significant correlations in the RD group, purple lines represent the significant correlations sharing by both groups. **(C)** Significantly larger inter-regional correlations between PAL and left IFG regions (the orange lines) in TD than RD group in the rhyming judgment task. No significantly larger subcortical-cortical correlations were found in RD than TD. The significant alpha level was set at *p* < 0.05 (FDR-corrected). RH, rhyming judgment task; ME, meaning judgment task; TD, typically developing group; RD, reading disabled group; L, left; R, right. See Abbreviations for full names of the abbreviations.

Figure 2 and Table 3 demonstrates significant inter-regional correlations between subcortical and classic cortical reading regions in the rhyming and meaning judgment tasks separately for the TD and RD group. Specifically, in the rhyming judgment task, both TD and RD showed significant inter-regional correlations between bilateral INS and left STG and between left INS and left IFGoperc. Additionally, the TD group showed significant inter-regional correlations between multiple subcortical regions (i.e., bilateral INS, left PAL) and left IFG regions. The RD group additionally showed significant inter-regional correlations between the right PUT and left STG (Figure 2A). Similar to the rhyming judgment task, in the meaning judgment task both TD and RD groups showed significant inter-regional correlations between left INS and left IFGoperc and between bilateral INS and left STG. The RD group additionally showed significant inter-regional correlations between the left PUT and left STG, and between the left PUT and two left IFG regions (Figure 2B). Taken together, the subcortical regions mainly connected to phonological processing regions (i.e., the left STG and IFG) in both the TD and RD groups for both tasks. Among those significant subcortical-cortical inter-regional correlations, the connectivity strength between the left PAL and the two left IFG regions was significantly larger in the TD than the RD group in the rhyming judgment task (as shown in Figure 2C and Table 4). No significantly larger inter-regional correlations were found in the RD than the TD group.

**Table 3:** Significant inter-regional correlations between subcortical regions and classic cortical reading regions in the rhyming and meaning judgment tasks separately for the TD and RD group.

**Task**	**TD**	***r***	***p***	**RD**	***r***	***p***
*RH*	**INS.L - IFGoperc.L**	0.428	<0.001	**INS.L - IFGoperc.L**	0.350	0.006
	INS.L - IFGtriang.L	0.342	0.005	**INS.L - STG.L**	0.533	<0.001
	**INS.L - STG.L**	0.518	<0.001	**INS.R - STG.L**	0.471	<0.001
	INS.R - IFGoperc.L	0.301	0.032	PUT.R - STG.L	0.321	0.032
	**INS.R - STG.L**	0.513	<0.001			
	PAL.L - IFGoperc.L	0.334	0.010			
	PAL.L - IFGtriang.L	0.371	0.005			
*ME*	**INS.L - IFGoperc.L**	0.364	0.003	**INS.L - IFGoperc.L**	0.380	0.002
	**INS.L - STG.L**	0.499	<0.001	**INS.L - STG.L**	0.519	<0.001
	**INS.R - STG.L**	0.481	<0.001	**INS.R - STG.L**	0.482	<0.001
				PUT.L - IFGoperc.L	0.324	0.030
				PUT.L - IFGtriang.L	0.290	0.038
				PUT.L - STG.L	0.295	0.038

*The alpha level for significant correlations was set at p < 0.05 (FDR-corrected). The connections in bold were significant subcortical-cortical correlations shared by both groups in each task. TD, typically developing group; RD, reading disabled group; RH, rhyming judgment task; ME, meaning judgment task; L, left; R, right. See Abbreviations for full names of the abbreviations*.

**Table 4:** Significantly larger inter-regional correlations between the left PAL and the left IFG regions in the TD than RD group in the rhyming judgment task.

**Inter-regional correlations**	**TD**	**RD**	***t***	***p***
PAL.L - IFGoperc.L	0.334	0.236	2.293	0.031
PAL.L - IFGtriang.L	0.371	0.212	2.445	0.044

*The alpha level for significant group difference was set at p < 0.05 (FDR-corrected). TD, typically developing group; RD, reading disabled group; L, left. See Abbreviations for full names of the abbreviations*.

### Brain-Behavior Correlation

Brain-behavior correlation between the inter-regional connections with significant group difference (i.e., the correlation between the left PAL and the two inferior frontal regions) and task performance of the rhyming judgment task for each group was calculated. We found significant positive brain-behavior correlations between the two inter-regional correlations and the respond time in the rhyming task in the RD group (the left PAL- the left IFGoperc: *r* = 0.626, *p* = 0.022; the left PAL-the left IFGtriang: *r* = 0.653, *p* = 0.015). As for the TD group, the same brain-behavior correlation showed an opposite tendency but didn't reach significant (i.e., the left PAL- the left IFGoperc: *r* = −0.092, *p* = 0.754; the left PAL-the left IFGtriang: *r* = −0.245, *p* = 0.398). And we found no significant correlations between the accuracy of the rhyming task and inter-regional correlations in either of the two groups.

## Discussion

In this study, we used graph theoretical analysis to examine the connectivity of subcortical regions within the reading network in typically developing (TD) and reading disabled (RD) Chinese children during two reading tasks. Two major findings emerged. First, reading disabled children engaged fewer subcortical hubs, and showed decreased subcortical-cortical connectivity compared to typically developing children. Second, alterations of the RD group in hub distribution and brain connectivity were mainly found in the rhyming judgment task compared to the meaning judgment task.

### Decreased Connectivity of Subcortical Regions in Chinese Children With Reading Disability

Perhaps the most interesting finding of the current study was that the RD group showed lower connectivity of subcortical regions than the TD group, particularly in the rhyming task, as demonstrated in hub distribution and inter-regional correlation analyses. When examining hub distribution, we found that multiple subcortical regions served as hubs in both groups across both tasks. However, subcortical regions were less involved within the RD group compared with the TD group. Specifically, the TD group had multiple unique hubs located in subcortical regions in both tasks (such as the bilateral thalamus, right putamen and left pallidum in the rhyming judgment task and the bilateral thalamus in the meaning judgment task). A hub is an essential node that is highly connected with other nodes within a network. Therefore, fewer subcortical regions serving as hubs in the RD group suggests less involvement of those subcortical regions during reading. When examining group differences in inter-regional correlations of the subcortical hubs, the left PAL in the RD group had weaker connections with the two left inferior frontal regions (i.e., the IFGtriang and IFGoperc) than those in the TD group during the rhyming judgment task. The left PAL has been suggested to be involved in motor process during speech articulation (Wise et al., 1999; Manes et al., 2014, 2018). The IFG, particularly the triangular and opercular regions, is also involved in speech articulation and phonological processing (Tan et al., 2005; Booth et al., 2007a; Cone et al., 2008; Wu et al., 2012). Thus, the weak connection between the left PAL and the inferior frontal regions may indicate a deficit in speech articulation, a process that lays the foundation for proficient reading (Shaywitz and Shaywitz, 2005; Rueckl et al., 2015). In addition, the correlations between the response time of the rhyming judgment task and the strength of these two connections showed opposite tendency between the two groups (i.e., positive in the RD group and negative in the TD group), indicating that these subcortical-cortical disconnections were related to the deficit in the phonological processing in the RD group. We assume that subcortical regions may play a role in “scaffolding”, allowing children to switch from sensory-motor based reading to abstract linguistic-based reading. This argument is consistent with previous findings that typical reading development is characterized by a shift from reliance on connections of subcortical regions to reliance on connections of cortical regions (Liu et al., 2018). Alternatively, the PAL is also closely connected with the striatum-frontal loop involved in procedural learning; the weaker connection between left PAL and the inferior frontal regions may instead indicate a deficit in procedural learning, and previous fMRI studies have implicated the cortico-striatal system in language learning (Bohland and Guenther, 2006; Booth et al., 2007b; Kita et al., 2013; Pugh et al., 2013). Taken together, these findings suggest that subcortical regions play an important role in typical development of Chinese reading, and that disconnection of the PAL with cortical phonological processing regions may be associated with a phonological processing deficit in Chinese dyslexic children.

These findings are generally consistent with previous studies showing subcortical abnormality in dyslexia [see Krishnan et al. (2016), for a review]. For example, a previous study found that people with dyslexia exhibited hyperactivation in the left caudate and thalamus during a rhyme judgment task using visual words (Hoeft et al., 2007). Other work has found that the left medial geniculate body of the thalamus was hypoactivated in adults with dyslexia compared to controls while performing an auditory processing task (Díaz et al., 2012). Siok et al. (2008) found that Chinese children with dyslexia showed hypoactivation in the left putamen and thalamus during a rhyming judgment task. Another study of functional connectivity found that Chinese children with dyslexia had weaker connections between the left putamen and the right inferior occipital gyrus, a visual orthographic processing region, during an auditory rhyming task (Wang et al., 2019), suggesting a deficit in linking orthography and phonology. In contrast, here we found weaker connections between another subcortical region (left PAL) with a traditional phonological processing region (IFG) in the Chinese children with reading disability in the visual rhyming judgment task, which may indicate a deficit in motor-based phonological processing neural system. The inconsistent findings between the study of Wang et al. (2019) and our study might be due to the two reasons. First, Wang et al. (2019) used an auditory rhyming judgment task, while we used a visual rhyming judgment task. The cognitive process involved in the rhyming judgment in the two modalities might be different. Second, the two studies used different data analysis approaches. Wang et al. (2019) firstly found reduced gray matter volume in the left putamen of the dyslexic group compared to the controls, so they further examined the role of this region in phonological processing by calculating functional connectivity from this region to other brain regions. But we compared the inter-regional functional connectivity between multiple subcortical regions and reading-related cortical regions. Despite of the inconsistent findings, both studies indicated essential roles of the subcortical regions in reading and the disconnection between subcortical regions and cortical regions might possibly be the neural basis of deficit in phonological processing in Chinese children with reading disability.

### RD Group Showed Larger Deficits in the Phonological Processing Than in the Semantic Processing

In terms of general hub distribution (as shown in Figure 1), the two groups shared more hubs in the meaning judgment task than in the rhyming judgment task. Specifically, the two groups shared only 33% of the hubs in the rhyming judgment task but 76% of the hubs in the meaning judgment task, suggesting that the two groups were more similar in the semantic processing than in the phonological processing to visual Chinese characters. In terms of subcortical hubs, significant group differences in inter-regional correlations were mainly found in the rhyming judgment task compared to the meaning judgment task (as shown in Figure 2; Table 3). These results suggest that phonological processing may be more deficient than semantic processing in the RD group from the perspective of connectivity. Further, although Chinese has a deep orthography, these results suggest that Chinese children with reading disability may have a deficit in accessing phonology from orthography. This finding is consistent with previous studies suggesting a phonological deficit in alphabetic dyslexia (e.g., Richlan et al., 2011) as well as in Chinese dyslexia (Siok et al., 2008; Cao et al., 2017). However, this finding is inconsistent with a previous study that reported brain activation abnormalities in Chinese children with dyslexia that were similar for both phonological and semantic processing (Liu et al., 2012). This inconsistency suggests that connectivity analysis may reveal subtler group differences than conventional brain activation analysis, leading to novel findings.

The finding of larger deficits in phonological vs. semantic processing in Chinese children with dyslexia might also be related to the properties of Chinese characters. Most Chinese characters are made of radicals, which can provide phonological or semantic clues. Semantic clues, which are often consistent with the meaning of the characters, can facilitate learning with respect to orthography to semantic mapping (Cao et al., 2010). However, phonological clues are often ambiguous, which makes learning the mapping between orthography and phonology much more difficult than learning the mapping between orthography and semantics (Shu, 2003). The accuracy data of the two tasks also showed that mapping from orthography to phonology was more difficult than mapping from orthography to semantic. That is, the nature of Chinese characters (i.e., consistent mapping between orthography to semantic vs. inconsistent mapping from orthography to phonology) may lead to a larger deficit in orthography to phonology mapping in Chinese children with dyslexia.

## Limitations

There are several limitations needed to be noted about our study. First, due to lacking a reading-level matched group, it is hard to address whether the alterations in the connectivity of subcortical regions in the RD group observed in the current study reflect poor reading experience or dyslexia itself. Further, the TD and RD groups were not perfectly matched as there were marginal differences in age and PIQ between the two groups. Although we had involved these two variables as control variables when performing the group difference analysis to reduce their potential influence, it's impossible to eliminate all the interference to the results. Second, the sample size of the current study is relatively small, which might limit the generalization of our findings. Third, the signal extraction method used in this study might induce some kind of collinearity problem, although we used jitter design trying to reduce its influence. Taken together, future studies are needed with a larger sample size, particularly with a reading-level matched group. In addition, more sophisticated time-series extraction method shall be used to reduce collinearity problem in future large-scale brain network studies.

## Conclusion

Subcortical, like cortical regions, can play a “hub” role in reading process of typical as well as atypical children readers. Reading impairment in Chinese is associated with less engagement of subcortical hubs and the disconnection between the subcortical hub (i.e., PAL) and the cortical region involved in speech articulation (i.e., IFG), suggesting a deficit in linking the subcortical sensory-motor system with cortical linguistic system. These alterations in hub distribution as well as in functional connectivity are more obvious in phonological than semantic processing to Chinese characters, suggesting the major deficit of Chinese children with reading disability lies in accessing and manipulating the phonology rather than meaning of the visual characters.

## Data Availability Statement

The original contributions generated for the study are included in the article/Supplementary Material, further inquiries can be directed to the corresponding author.

## Ethics Statement

The studies involving human participants were reviewed and approved by the Institutional Review Board at Beijing Normal University. Written informed consent to participate in this study was provided by the participants' legal guardian/next of kin.

## Author Contributions

LZ analyzed the data and wrote the manuscript. JH collected and pre-processed the data. XL collected the data and helped analyse the data. EN and CL revised the manuscript. LL designed the experiment and wrote the manuscript. All authors contributed to the article and approved the submitted version.

## Conflict of Interest

The authors declare that the research was conducted in the absence of any commercial or financial relationships that could be construed as a potential conflict of interest.

## Footnotes

^1^http://rfmri.org/DPARSF

^2^http://www.nitrc.org/projects/gretna

## Abbreviations

ACG: anterior cingulate and paracingulate gyri
ORBinf, inferior frontal gyrus: orbital part
CAU: caudate nucleus
ORBsupmed, superior frontal gyrus: medial orbital
DCG: median cingulate and paracingulate gyri
PAL: pallidum
FFG: fusiform gyrus
PCG: posterior cingulate gyrus
IFGoperc, inferior frontal gyrus: opercular part
PUT: putamen
IFGtriang, inferior frontal gyrus: triangular part
REC: gyrus rectus
INS: insula
ROL: rolandic operculum
IOG: inferior occipital gyrus
SFGdor, superior frontal gyrus: dorsolateral
IPL: inferior parietal lobule
SFGmed, superior frontal gyrus: medial
ITG: inferior temporal gyrus
SMA: supplementary motor area
LING: lingual gyrus
SPG: superior parietal gyrus
MOG: middle occipital gyrus
STG: superior temporal gyrus
MTG: middle temporal gyrus
THA: thalamus.
