# Supplementary material for: Disrupted Subcortical-Cortical Connections in a Phonological but Not Semantic Task in Chinese Children With Dyslexia
Source: Front Hum Neurosci. 2021 Jan 18;14:611008. doi: 10.3389/fnhum.2020.611008 (PMC7848143; doi:10.3389/fnhum.2020.611008)
Supplement: Supplementary file 1 [file Table_1.DOCX]

Supplementary Material

Supplementary Table 1

Demographics and task performance of the typically developing (TD) and reading disabled (RD) groups in rhyming judgment task.

|  | TD | RD | p |
| --- | --- | --- | --- |
| Gender(M/F) | 11/5 | 9/5 | 0.804 |
| Age (mean (SD)) | 11.70(0.39) | 12.06 (0.51) | 0.103 |
| Performance IQ | 111(15) | 102(10) | 0.086 |
| CRM | 2991 (157) | 2548(87) | <0.001 |
| CRF | 1.06(0.21) | 0.62(0.15) | <0.001 |
| Rh_ACC (mean (SD)) | 73% (0.12) | 58% (0.10) | <0.001 |
| Rh_RT (mean (SD)) | 1755(445) | 1376 (390) | 0.020 |
| Me_ACC (mean (SD)) | 82% (0.10) | 71% (0.10) | 0.006 |
| Me_RT (mean (SD)) | 1492 (394) | 1424(334) | 0.617 |

Note: TD: typically developing group, RD: reading disabled group, CRM: Character Recognition Measure and Assessment Scale for Primary School Children, CRF: Character Reading Fluency test. Rh_ACC and Rh_RT: the accuracy and reaction time (ms) in character condition of the rhyming judgment task; Me_ACC and Me_RT: the accuracy and reaction time in character condition of the meaning judgment task.

Supplementary Table 2

Group unique and sharing subcortical hubs.

|  | Hubs | Tasks |
| --- | --- | --- |
| TD unique hubs | Bilateral THA | RH&ME |
|  | PUT.R | RH |
|  | PAL.L | RH |
| TD & RD sharing hubs | Bilateral INS | RH&ME |
|  | PUT.L | RH&ME |
|  | Bilateral PAL | ME |
|  | PUT.R | ME |

Note: TD, typically developing group; RD, reading disabled group; RH, rhyming judgment task; ME, meaning judgment task; L, left; R, right. There were no RD unique subcortical hubs.
